# P-424. Utility of Plasma Microbial Cell-Free DNA Testing via the Karius Spectrum Test in Clinical Decision Making in Children

**DOI:** 10.1093/ofid/ofaf695.640

**Published:** 2026-01-11

**Authors:** Vincent Anella, Jared Olson, Jena Rhodes, Sonia Mehra, Adam Hersh, Emily A Thorell, Andrew T Pavia, Elizabeth D Knackstedt, Anne J Blaschke

**Affiliations:** Intermountain Health, Milcreek, UT; Intermountain Health, Milcreek, UT; Intermountain Health, Milcreek, UT; University of Utah, Salt Lake City, Utah; University of Utah, Salt Lake City, Utah; University of Utah, Salt Lake City, Utah; University of Utah, Salt Lake City, Utah; University of Utah, Salt Lake City, Utah; University of Utah School of Medicine, Salt Lake City, UT

## Abstract

**Background:**

Microbiologic diagnosis of infections improves treatment. Traditional culture-based methods have limitations including decreased yield in the setting of pretreatment, need for invasive sampling, and special media for detection of fastidious organisms. Cell-free DNA testing (cfDNA-T), such as the Karius Spectrum test, may mitigate these limitations. We describe the use of cfDNA-T in children and the impact by clinical condition.

**Table 1:**
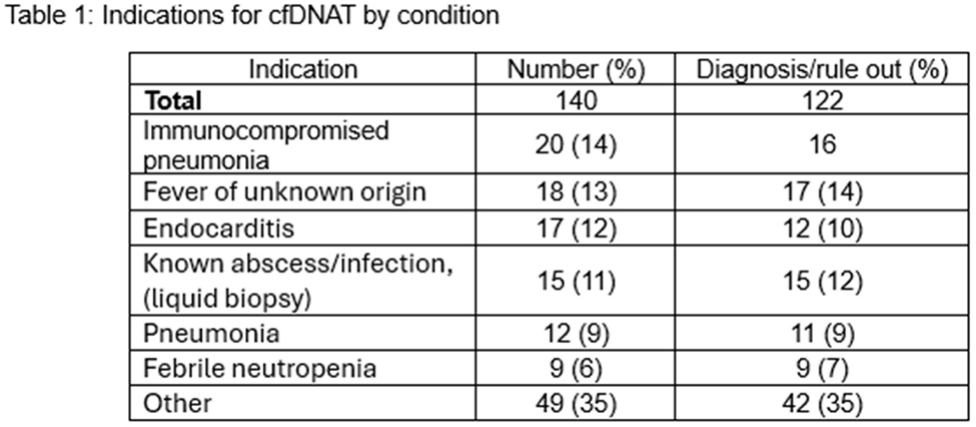
Indications for cfDNAT by condition

**Table 2:**
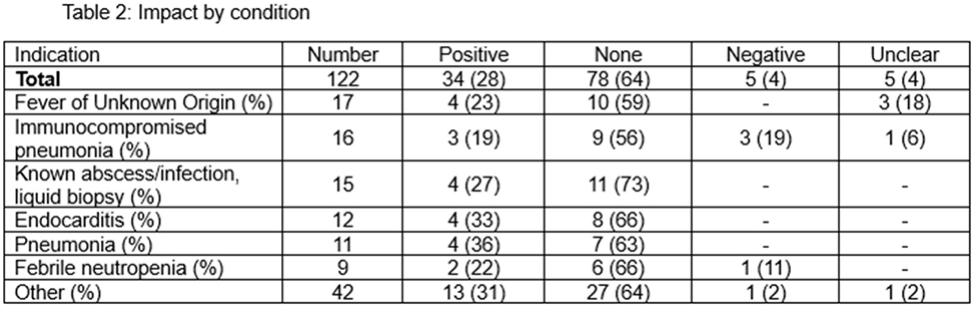
Impact by condition

**Methods:**

We included patients in the retrospective review who underwent cfDNA-T sent for the purpose of diagnosis between June 2020 and June 2024. All tests were approved by an infectious diseases clinician. We reviewed both the indication for conducting the test (clinical scenario) and categorized impact as having positive, negative, or no clinical benefit. Common clinical scenarios included endocarditis, febrile neutropenia, fever of unknown origin, known abscess/(liquid biopsy), pneumonia, and pneumonia in an immunocompromised patient (Table 1).

**Table 3:**
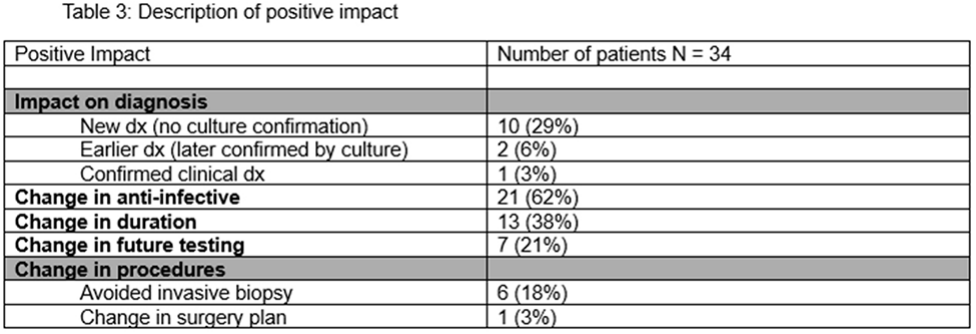
Description of positive impact

**Results:**

We reviewed at total of 122 tests on 112 patients conducted to establish a diagnosis of which 69 of 122 (57%) were positive for at least 1 organism. Yield was highest in pneumonia (36%) and endocarditis (33%).cfDNA-T had a positive clinical impact in 34/122 (28%) cases, with 21/34 (62%) resulting in antimicrobial changes. In 10 cases (8%), cfDNA-T led to a diagnosis that would not have been made another way (Table 3). Negative clinical impact occurred in 5/140 cases (3.5%) resulting in unnecessary treatment (2 patients), additional tests (2), and longer inpatient stay (5).

**Conclusion:**

When used for diagnosis, cfDNA-T identified a pathogen in 28% and led to a positive clinical benefit in 28% of patients. Changes to antimicrobial therapy were the most frequent positive clinical impact. Prospective studies are needed to optimize the use of this diagnostic tool.

**Disclosures:**

Andrew T. Pavia, MD, Antimicrobial therapy inc.: Royalties|Haleon: Advisor/Consultant Anne J. Blaschke, MD, PhD, BioFire Diagnostics/Biomerieux: I have IP owned by the U. of Utah licensed to BioFire and receive royalties.|Merck & Co: Advisor/Consultant

